# Welfare Impact of Community-Based Veterinary and Breeding Services on Small Ruminant Keepers

**DOI:** 10.3389/fvets.2021.610610

**Published:** 2021-07-29

**Authors:** Girma Tesfahun Kassie, Woinishet Asnake, Aynalem Haile, Tesfaye Getachew Mengistu, Solomon Gizaw, Barbara Rischkowsky

**Affiliations:** ^1^International Center for Agricultural Research in the Dry Areas (ICARDA), Rabat, Morocco; ^2^International Center for Agriculture Research in the Dry Areas (ICARDA), Addis Ababa, Ethiopia; ^3^International Livestock Research Institute (ILRI), Addis Ababa, Ethiopia

**Keywords:** difference-in-differences, Ethiopia, community based breeding, veterinary services, JEL: C18, C21, Q12, Q13

## Abstract

Improved breeding practices and participatory health services have been designed and implemented by a partnership between national and international institutions in various parts of Ethiopia since 2014. Based on a panel data of two waves, we have estimated the impact of these interventions on small ruminant fertility, offtake, return per head of animal, and gross income per adult equivalent. Different specifications of the difference-in-differences model revealed that access to small ruminant health services has increased offtake, return per head of sheep/goat, and gross income per adult equivalent. Participants in community-based small ruminant breeding have also higher offtake and gross income per capita than those who are not taking part. The findings of this study are expected to help understand the economic benefits that accrue to rural areas when livestock development interventions are made based on the right diagnosis. The results of this study will also be useful in informing the ongoing discussion in Ethiopia on the transformation of the livestock sector.

## Introduction

Livestock are a crucial part of the rural livelihoods in many developing countries where global and local challenges are making the effort to reduce food security and worsen poverty. In Ethiopia, an East African country with an estimated human population of 110 million, rural communities eke out a living from a structurally and institutionally constrained extensive agricultural system. The pastoral and dry lowland parts of the country inhabit communities that depend entirely on livestock for their livelihoods. In the midlands and highlands, crop–livestock mixed production systems are the mainstay of the rural economy. The national herd—consisting of about 55.2 million cattle, 29.3 million sheep, 29.1 million goats, 4.5 million camels, and close to 50 million poultry—sustains, at least partially, the livelihoods of more than 11.3 million rural households (1, 2).

Small ruminants have a multidimensional contribution to the smallholder farmers' livelihoods including economic, social, nutritional, and environmental benefits (3). Subsistence farmers prefer sheep and goats, as the risk of losing large ruminants is often remarkably high (4). Sheep and goats are the best options to improve food security and diversify household livelihood strategies, as they require lower initial capital investment and other production resources such as land and feed. Mainly kept as store of value and as readily available liquid assets, the production and market performance of small ruminants has a clear implication on the financial viability of the smallholder farm households.

The production and productivity of small ruminants have, however, been reported to be low mainly because of poor genetics of the sheep and goat population, high disease incidence and parasite challenges, and lack of feed and forages (5–7). Similarly, the marketing of small ruminants has hardly been rewarding to small ruminant keepers because of inadequacy or absence of market information, market infrastructure, market orientation, and policy support (1).

There have been several efforts to improve the genetics of the indigenous sheep and goat populations (8–10). Nonetheless, sheep and goat breeding strategies in Ethiopia focused on importing exotic breeds. Different governmental (research and academic) and non-governmental institutions and projects implemented these introductions and crossbreeding (11). These programs generated no significant effects on sheep and goat productivity or on farmers and pastoralists' livelihoods and the national economy at large. The major limitations faced have been the lack of a clear breeding and distribution strategy, little consideration of the needs of the farmers and pastoralists, limited or no participation in the design and implementation of the breeding programs, and the lack of schemes to sustain crossbreeds at the village level (7, 11).

Similarly, although there have been decades-old interventions to improve the accessibility and quality of animal health services, the overall achievement has hardly been commendable. Animal diseases affect the livestock population in Ethiopia in many ways including slow growth, low fertility, mortality, and morbidity. The annual loss due to mortality ranges from 8 to 10% for cattle, 12 to 14% for sheep, 11 to 13% for goats, and 56.9% for poultry (12). The major small ruminant health interventions are vaccination and ectoparasite control. Major achievements in vaccination is *peste des petits* ruminants (PPR) vaccination (13). Ectoparasite control efforts were introduced through community-based non-governmental organizations (NGOs) into pastoral areas (14).

The current delivery of animal health services is inadequate both in coverage and in quality. Only 45% of the country is served with animal health delivery systems (12). Alemu et al. (15) argued that animal health research and development interventions tend to deal with animal diseases that affect trade, are transboundary in nature, or are zoonotic. Even though these diseases potentially play a key role in adversely affecting food security and the livelihood of smallholder farmers, little work has been done on endemic diseases, and their contribution to loss of productivity is poorly documented (16).

Since 2012, a new global partnership under the CGIAR's Livestock and Fish Research Program (Livestock Research Program since 2017) initiated and implemented more participatory and local knowledge-based approaches in small ruminant health and breeding programs in Ethiopia. These approaches identified interventions based on comprehensive characterization of the small ruminant production systems in the intervention sites. The interventions involved national partners and individual farmers with the purpose of increasing productivity and financial returns from the livestock (9, 10).

Our research started with the hypothesis that improved veterinary services and breeding practices affect small ruminant fertility and offtake and then improve returns per head of animal and gross income per capita. To assess these impacts, two rounds of comprehensive surveys were conducted in 2014 and 2018. This paper reports the findings of an empirical analysis of the effect of these interventions on selected immediate and long-term outcomes. Using panel data treatment effect models, we report that access to veterinary services improved market participation in terms of increased offtake, income earned per head of sheep and goat, and gross income per adult equivalent. Similarly, taking part in small ruminant breeding programs improved offtake and gross income per adult equivalent. The interventions happened to have no statistically significant effect on the number of lambs/kids per the total number of breeding age does/ewes in a year. The positive effects need to be seen within the context of the crucial role that small ruminants play in the livelihoods of rural communities in Ethiopia.

This study contributes to the relevant body of knowledge in at least three ways. First, we are not aware of any other study in Ethiopia or in sub-Saharan Africa that evaluated the welfare impact of community-based breeding and veterinary services on small ruminant keepers. Given the size of the small ruminant population and the heterogeneity of the production systems in the country, the findings of this study will have relevance to a broader research and development community. Second, we hope that the empirical evidence on the average effect of the community-based breeding and veterinary interventions informs breeders and animal health practitioners on the economic implications of the efforts they are exerting. Finally, the research will also inform policymakers on the importance of and justification for the investment in community-based breeding programs and veterinary services for small ruminants. Considering the insufficient attention given to the small ruminant value chains in the country, this information is expected to help in revising the prioritization of the different livestock development interventions.

## Materials and Methods

### Description of the Interventions

Data-intensive advanced breeding programs or introduction of live animals for cross breeding could hardly be implemented in Ethiopia with the required level of complexity or expected level of success (10, 17). This observation gave rise to a different approach for small ruminant breeding. The novel approach, called community-based breeding program (CBBP) was started in 2009 with four sheep breeds (*Afar, Bonga, Horro*, and *Menz*) representing different production systems and involving eight communities in Ethiopia (10). These pilot CBBPs have since expanded to include more than 40 communities and have also been introduced to other countries including Burkina Faso, Iran, Liberia, Malawi, South Africa, Sudan, Tanzania, and Uganda. CBBP is a better option compared with the conventional nucleus schemes or importation of exotic breeds in that it is inherently sustainable as it supports local-level decision making, focuses on locally adapted indigenous breeds, and considers the constraints that smallholder farmers face (10, 18). CBBP involves collective action, participatory breeding goal definition and trait identification, breeding male selection, distribution of selected sires and introducing mating management, culling of unselected males, training of farmers, and data collection and management (Table 1).

**Table 1:** Description of the components of the breeding innovation.

**Component**	**Description**
• Breeders' cooperatives and controlled small-ruminant mating groups	° In each site, breeders' cooperative and different mating groups were organized. Cooperatives facilitate regular animal identification, data collection and recording, sire use, and management and rotation among mating groups.
• Definition of breeding objectives and selection traits	° Identification of the reasons why farmers/pastoralists keep their animals and the attributes they value most is crucial in breeding programs.
• Ranking and selection of best breeding males	° At the beginning, sires were ranked based on their genetic worth (estimated breeding values) for agreed breeding objective traits and farmers selection criteria.
• Transfer/dissemination of improved sires to the participants and arrange mating system	° Culling of older/unfit sires and dissemination of new as replacement done once [in the other sites] per year focusing on replacing older sires. This ensures that all flocks have enough and good quality breeding sires to mate their breeding females.
• Awareness creation, field day, and training on small ruminant breeding techniques and capacity development	° This involves workshop and field days aiming at sharing experiences, and training of participating breeders, extension workers, and researchers. °Pregnancy test using ultrasound, fresh semen collection, and artificial insemination started in some of the sites. Field artificial insemination facilities put in place in all CBBP sites.
• Culling and selling of non-selected males	° Older sires have been culled, fattened, and sold in good price for meat.
• Monitoring and evaluation	° Data collection and animal identification have been checked and evaluated. Data collected on performances have been analyzed and used to check the genetic progress for traits of interest.
• Certification of improved genetics	° Breeding sires need to be certified for genetic merit, reproductive performance, and reproductive diseases. This enables dissemination of improved genetics to the base population.
• Establishment of reproductive platforms	° Establishing reproductive platform was identified to be key for fertility improvement and dissemination. The platform assists in mass estrus synchronization, artificial insemination, and pregnancy diagnosis using ultrasound.
• Development of suitability maps for sheep and goats	° Mapping breeds/population to suitable environments is important in planning livestock breeding and scaling activities due to its efficiency in allocating improved and new breeds to appropriate habitats for optimal production. In the context of predicting suitable habitats for selected breeds of indigenous Ethiopian sheep and goats, we used geo-informatics based spatial analytic tools to develop breed-specific suitability index maps.

*CBBP, community-based breeding program*.

The breeding interventions were undertaken across locations in various parts of the country. Sheep breeding programs have been implemented in *Menz, Horro*, and *Doyo gena* districts. Goat genetic improvement interventions were undertaken in *Abergelle* district. *Doyo gena, Horro*, and *Menz* represent sheep-dominated production systems. *Abergelle* represents goat-dominated production systems. We combined the two species, and hence, we will refer to the interventions as small ruminant breeding practices. In each of the districts, there are intervention and control *Kebeles*^1^. We considered farmers who were trained and who understood and practiced the different components of the breeding programs in the intervention sites as participants of the improved breeding program.

Animal health interventions were introduced into the study sites as part of the concerted effort to transform the small ruminant value chains. Participatory epidemiological approach (19), was adopted and veterinary health interventions were developed and embedded in the CBBPs. The key assumption behind the choice of this community-based approach is that prevention of selected infectious and non-infectious diseases is less expensive than treating conditions as they occur (20). The design of the interventions was guided by participatory identification and prioritization of the diseases of sheep and goat (15, 21).

The projects districts are *Abergelle, Menz*, and *Doyo gena* with adjacent intervention and control *Kebeles* within each district. The health interventions included strategic vaccination for different respiratory diseases, control of reproductive diseases, and deworming for gastrointestinal parasites in small ruminants (Table 2) (11, 20, 22). As there are different health service providers in the districts, we considered farm households in the intervention areas who received the services (presented in Table 2) only from the formal extension system or the research centers as participants (treatment group) and the rest as non-participants (control).

**Table 2:** Description of the components of the small ruminant health intervention.

**Component**	**Description**
Deworming SR for gastrointestinal parasites and lungworms	Intended to reduce worm burden in the small ruminant population.
Training farmers on control of SR gastrointestinal parasitosis	Training for farmers on transmission cycles and principles of parasite control
Vaccination for key production diseases	Site-specific vaccination campaigns on ovine pasteurellosis, *peste des petits* ruminants, sheep, and goat pox.
Training of farmers on control of SR respiratory diseases	Training sessions on respiratory diseases and how to control them to ensure vaccinations had the desired buy-in of farmers.

### Sampling

This study used a combination of purposive and random sampling. The study districts were selected with the purpose of developing benchmarks for the interventions of the global research initiative on small ruminant value chain development—which Ethiopia is part of. First off, the intervention and control *Kebeles*^2^ were identified. Then, the list of households in the sample *Kebeles* was developed from health service roster or that of taxpayers. Then, we identified households using the lottery method with replacement from each district proportional to the district population size. In total, the study covered nine districts where 1,108 households were visited in 28 *Kebeles*. The sample for the baseline can be considered as representative of the smallholder producers in the country.

The end line survey in 2018 covered only sites where the small ruminant health and improved breeding interventions have been ongoing since 2014^3^. The end line covered *Menz* and *Abergelle* in Amhara Region, *Horro* in Oromia Region, and *Doyo gena* in Southern Nations, Nationalities, and People's Region (SNNPR).

For the end line survey, we talked to the participants and non-participants that we visited in the baseline survey in these four districts. In total, we talked to 571 farm households with an attrition rate of only about 5%. We found 29 observations to be incomplete and, hence, dropped them from the analysis. Therefore, we have a balanced panel of 542 observations for the analysis reported in this article.

The respondents in the survey are household heads or representatives of the household head. We considered fathers and mothers of the house as household heads and talked to whoever was available for the interview. The objective of the study is estimating impact at the household level, and hence the household was the unit of data generation and analysis.

### Econometric Framework

We have four outcomes that we hypothesized to be affected by the interventions discussed above. The first outcome is fertility. Fertility is measured in terms of the number of lambs or kids per a breeding ewe or doe in a year. The second outcome variable is offtake measured in terms of the number of sheep or goat sold within a year per household. The third is the average price received per head of animal sold in birr^4^ (return/animal). The fourth outcome variable is gross annual household income (income) in birr per adult equivalent (AE).

Estimating the impact of the small ruminant health and improved breeding interventions on our outcomes of interest (fertility, offtake, return/animal, and income/AE) entails comparing the observed outcomes with the outcomes that would have resulted had the smallholders never accessed the interventions. However, the farm households are either participating or not participating, and, hence, we cannot observe both outcomes in the two states of nature (23, 24). Yet identification of the effect of the interventions on the outcome variables requires development of a meaningful counterfactual, i.e., the potential outcome of farmers who participated had they not participated at all.

One of the most common analytical frameworks employed to identify cause-and-effect relationships in a panel data setting is difference-in-differences (DiD) (24, 25). The DiD model is considered as an alternative estimation strategy to deal with possible selection bias by controlling time-invariant differences between treatment and control groups (26–28). In addition, as it can be combined with some other procedures, such as propensity score matching (PSM), the method is a more flexible form of causal inference than other non-experimental methods (29). In this study, therefore, we have employed different specifications of DiD model to estimate the average treatment effect (ATE) on small ruminant fertility, offtake, return per animal, and gross income per adult equivalent of smallholder farmers due to accessing or participating in community-based veterinary or breeding interventions.

DiD estimates the treatment effect based on the data collected from the treatment [accessing/participating] and control [non-accessing/non-participating] groups before and after the intervention. Since the DiD assumes that unobserved heterogeneity in participation is present but is constant over time, it resolves the problem of missing data (unobserved heterogeneities) by differencing out the constant components and provides a more robust estimate of the impact of treatment on participants (25, 30).

The ATE is by definition the difference between the expected values of the differences of the outcomes observed over the two periods conditional on the treatment level. Given a two-period panel setting (*t* = 0, 1), where *t* = 0 refers to before the interventions or baseline and *t* = 1 after the interventions or end line, and the outcome variable for participants is $Y_{t}^{p}$ and non-participants is $Y_{t}^{n}$ in time *t*, ATE of the intervention (T) using DiD can be estimated by

(1) $$\begin{array}{r} \delta =E\left(Y_{1}^{p}-Y_{0}^{p}|T_{i}=1\right)-E\left(Y_{1}^{n}-Y_{0}^{n}|T_{i}=0\right) \end{array}$$

where *δ* denotes DiD and *T*_*i*_ is a treatment indicator equal to 1 if the household is a participant and 0 otherwise.

The DiD can also be estimated within a fixed-effects (FE) regression framework. DiD makes a similar assumption with FE model, but conditions on a group level instead of an individual level effect (31, 32). Following Ravallion (30) and Chakrabarti et al. (26), the DiD model can be specified as an FE linear regression model:

(2) $$\begin{array}{r} Y_{it}=\alpha +\rho T_{i1}+\gamma t+\beta T_{i1}t+\epsilon_{it} \end{array}$$

where *Y*_*it*_ is an outcome measure of household *i* at time *t* and *ϵ*_*it*_ is the error term, which includes all unobserved determinants of *Y*_*i*_ not included in the model. *α* is a constant term, *ρ* denotes specific effect of treatment group (to account for the average unobserved difference between participating and non-participating households which is constant over time), and *γ* denotes the effect of time FEs. The coefficient β represents the effect of the interaction of treatment and time and hence gives the average DiD effect.

The FE model discussed above is robust to some forms of endogeneity arising from unobservable treatment-specific heterogeneity (31). Specifically, FE models allow covariates to be endogenous provided that they are correlated only with a time-invariant part of the error (33). DiD, as a form of two-way FE model, can control both observed and unobserved heterogeneity (34). More specifically, the outcome variable *Y*_*it*_ can be regressed on treatment status *T*_*it*_, a range of time-varying covariates *X*_*it*_, and unobserved time-invariant individual heterogeneity *η*_*i*_ that may be correlated with both the treatment and other unobserved characteristics *ϵ*_*it*_. Hence, the FE model of Equation (2) can be rewritten as

(3) $$\begin{array}{r} Y_{it}=\phi T_{it}+\delta X_{it}+\eta_{i}+\epsilon_{it} \end{array}$$

Differencing both sides of Equation (3) over time, one would obtain the following equation:

(4) $$\begin{array}{l} \left(Y_{it}-Y_{it-1}\right)=\phi \left(T_{it}-T_{it-1}\right)+\delta \left(X_{it}-X_{it-1}\right)\\ \;+\left(\eta_{i}-\eta_{i}\right)+(\epsilon_{it}-\epsilon_{it-1}) \end{array}$$

(5) $$\begin{array}{r} \text{Δ}Y_{it}=\phi \text{Δ}T_{it}+\delta \text{Δ}{\text{X}}_{\text{it}}+\text{Δ}\epsilon_{it} \end{array}$$

Since the source of endogeneity (the unobserved individual characteristics *η*_*i*_) has dropped due to differencing, ordinary least squares (OLS) can be used to estimate the unbiased effect of the intervention (*ϕ*). With two time periods, *ϕ* is equivalent to the DiD estimate in Equation (2) above.

For DiD to yield an unbiased estimate of causal impact, the key assumption of DiD, i.e., the parallel trend assumption, should hold (35). However, it is possible that the initial conditions of intervention and control areas are not similar in terms of some observed and unobserved characteristics in which the subsequent outcome changes might be a function of this difference, which may confound the result (28, 36). The presence of time-varying heterogeneity associated with selection into the treatment groups may cause the parallel trend assumption to be violated and bias DiD estimates (30, 37).

Controlling for initial treatment specific conditions can be used to resolve the effect of time-varying factors that might bias the estimate. In our study, the treatment assignment is not correlated with the error terms of the model. However, the initial conditions may have a separate effect on the changes in outcome as well. We are, therefore, combining PSM and DiD not only to deal with endogeneity that might arise from omitted variables but also to control for all other sources of variation at the start of the study (38). This virtue of combining the two estimators emanates from the fact that PSM is non-parametric, helps balance covariates, and creates a more focused causal inference (25, 30). Hence, using a two-period data of accessing/participating and non-accessing/non-participating groups, the propensity score was used to match participant and control units in the base period, and then the treatment impact was calculated using DiD to the matched sample. Following Guo and Fraser (39), with panel data over two time periods, *t* = {0, 1}, DiD estimator for the mean difference in outcomes *Y*_*it*_ for each treatment unit *i* is given by

(6) $$\begin{array}{r} \delta_{i}=\left(Y_{i1}^{p}-Y_{i0}^{p}\right)-\underset{j\in c}{\sum }w\left(i,j\right)\left(Y_{j1}^{n}-Y_{j0}^{n}\right) \end{array}$$

where *ω*(*i, j*) is the weight (based on the propensity score) attached to each control unit *j* matched to treatment unit *i*. Hence, to ensure the robustness of the ATE estimates, we have estimated the linear FE and DiD with PSM models.

## Results and Discussion

### Description of the Sample Households

We briefly describe the sample households comparing them between intervention and control sites. Our sample was composed mainly [83.8%] of male respondents. The respondents were on average 47 years of age with education of only 1.29 years. Average literacy, in number of years, has slightly gone down in the control sites in 2018, and, yet on aggregate, literacy is higher in control areas than intervention sites. The average household size of the sample respondents was close to six individuals, which is equivalent to the national average for midlands and highlands (40).

The average distance to livestock markets, measured in kilometers [km], is 6.02 for the entire sample. The distance is slightly shorter for the sample in the intervention sites where there is a considerable drop between 2014 and 2018 (Table 3). More than 66% of the sample respondents had contacts with agricultural extension agents in relation to small ruminant production. Access to small ruminant-related extension services is lower in control areas even if there is more pronounced leap between 2014 and 2018 in these areas.

**Table 3:** Summary statistics of sample households by survey period and treatment status.

**Variable**	**Unit**	**Baseline [2014]**		***N***		**End line [2018]**		***N***		**Pooled**			***N***	
		**Cont. site**	**Interv. site**		**Sig**.	**Cont. site**	**Interv. Site**		**Sig**.	**Cont. site**	**Interv. site**	**Total**		**Sig**.
Gender of respondents (1 = male)	%	87.0	82.2	542		83.3	83.1	542		85.2	82.7	83.8	1,084	
Age (years)	^#^	44.87 (0.89)	46.06 (0.90)	540	NS	46.91 (0.96)	49.59 (0.86)	542	^*^	45.87 (0.65)	47.86 (0.63)	47.00 (0.46)	1,082	^*^
Education (year)	^#^	1.31 (0.07)	1.29 (0.06)	540	NS	1.22 (0.07)	1.34 (0.07)	541	NS	1.26 (0.05)	1.32 (0.05)	1.29 (0.03)	1,081	NS
Family size [count]	^#^	6.72 (0.14)	6.22 (0.12)	542	^**^	6.68 (0.15)	6.16 (0.12)	542	^**^	6.7 (0.10)	6.19 (0.08)	6.41 (0.07)	1,084	^^**^*^
Distance to market	km	6.59 (0.39)	6.27 (0.75)	451	NS	6.72 (0.37)	4.86 (0.26)	448	^**^	6.65 (0.27)	5.55 (0.39)	6.02 (0.25)	899	^*^
Access to extension (1 = yes)	%	55.2	70.6	542		62.3	73.9	542		58.7.	72.3	66.4	1,084	

*Standard deviation in brackets*.

* *p < 0.10*,

** *p < 0.05*,

*** *p < 0.01*,

# *Number*.

*Cont. denotes control sites, Interv. denotes intervention, Sig. denotes statistical significance (>0) of the mean difference between control (non-participants) and intervention (participants), and NS denotes not significant*.

Mean comparison between the samples in the two sites over the two waves of survey shows that only the difference in family size is statistically significant (*p* < 0.01) at the baseline level. In the end line survey, however, we noted statistically significant differences around age of the respondent (*p* < 0.05), family size (*p* < 0.01), and average distance from the market in kilometers (*p* < 0.001).

### Summary of the Outcome Variables

There is clear difference between intervention sites and control sites in the initial level of small ruminant fertility rate. The gap, however, remains to be comparable between the two waves (Table 4). The other variable with considerable difference between the samples in the two sites is total number of sheep and goat sold over a period of 12 months (offtake). In 2014, the offtake level in control sites is nearly twice that of the intervention sites. In 2018, the offtake in the intervention sites has increased to the extent that it is higher than the level in control sites (Table 4). The other outcome variables do not show any peculiar difference over the two periods between the two sites.

**Table 4:** Summary statistics of outcome variables.

**Variable**	**Unit**	**Baseline [2014]**		***N***		**End line [2018]**		***N***			**Pooled**		
		**Cont. site**	**Interv. site**		**Sig**.	**Cont. site**	**Interv. Site**		**Sig**.	**Cont. site**	**Interv. site**	***N***	**Sig**.
Fertility rate of the herd	#	25.89 (2.61)	31.34 (2.62)	502	NS	26.98 (2.48)	31.24 (2.38)	518	NS	26.42 (1.8)	31.29 (1.76)	1,020	NS
Total sheep and goat offtake	#	9.31 (1.28)	5.47 (0.92)	526	^*^	22.66 (1.66)	23.09 (1.31)	519	NS	15.7 (1.09)	14.46 (0.88)	1,045	NS
Ln(return/head)	#	4.42 (0.12)	4.29 (0.21)	107	NS	4.67 (0.08)	4.77 (0.06)	377	NS	4.6 (0.07)	4.69 (0.06)	484	NS
Ln(income/AE)	#	7.69 (0.08)	7.91 (0.06)	542	^*^	8.19 (0.07)	8.10 (0.06)	542	NS	7.93 (0.05)	8.01 (0.04)	1,084	NS

*Standard deviation in brackets*.

* *p < 0.10*,

** *p < 0.05*,

*** *p < 0.01*.

*Cont. denotes control sites, Interv. denotes intervention, Sig. denotes statistical significance (>0) of the mean difference between control (non-participants) and intervention (participants), and NS denotes not significant. Ln(return/head) denotes natural log of revenue generated per head of sheep/goat in birr. Ln(income/AE) denotes natural log of gross annual income per adult equivalent in birr*.

*AE, adult equivalent*.

Simple mean comparisons show that there were no statistically significant differences between the two groups of farm households in 2018 and over the pooled data. In the baseline, however, the differences between the two groups in terms of total sheep and goat offtake and logarithm of total reported income per AE were statistically significant (*p* < 0.1).

### Econometric Results

We report three sets of causality models in this section. The first set is DiD estimations using FE regression with no other covariates (Equation 2). The second set is DiD estimations using FE regression with time variant other covariates (Equation 5). Finally, the third set is combination of PSM and DiD models to control for initial conditions of the sample respondents and compare only those households with comparable likelihood of participation (Equation 6).

The estimation that compares participants and non-participants shows that the access to veterinary services and improved sheep and goat breeding practices significantly increases offtake at the household level. This estimator does not consider any confounding factors and still shows that households with access to veterinary services have supplied six more small ruminants to the market over a period of 12 months. Similarly, farm households who participated in CBBP have on average supplied nine more sheep/goat over a year as compared with those who did not participate (Table 5). This model resulted in insignificant cause-and-effect relationship between the other three outcome variables.

**Table 5:** DiD with basic fixed-effects specification without covariate.

	**Fertility**		**Offtake**		**Ln(return/head)**		**Ln(income/AE)**	
	**Model 1**	**Model 2**	**Model 1**	**Model 2**	**Model 1**	**Model 2**	**Model 1**	**Model 2**
Animal health ^*^ Year	−2.56 [−0.44]		5.94^**^ [2.40]		0.54 [1.21]		−0.19 [−1.56]	
Breeding ^*^ Year		−5.94 [−1.00]		8.88^***^ [3.37]		0.27 [0.67]		−0.18 [−1.40]
Year	1.60 [0.48]	0.41 [0.12]	13.47^***^ [8.47]	13.32^***^ [8.80]	0.25 [1.00]	0.27 [1.22]	0.44^***^ [5.07]	0.43^***^ [5.14]
Animal health	0.99 [0.15]		−5.22^**^ [−2.23]		−0.19 [−0.42]		−0.12 [−0.80]	
Breeding		12.96^**^ [2.00]		−7.78^***^ [−3.19]		0.24 [0.53]		−0.09 [−0.70]
Constant	28.62^***^ [9.48]	25.68^***^ [11.23]	9.12^***^ [8.84]	9.12^***^ [11.10]	4.32^***^ [18.85]	4.23^***^ [24.44]	7.86^***^ [119.27]	7.84^***^ [163.06]
*N*	1,020	1,020	1,045	1,045	484	484	1,084	1,084
*N*_cluster	544	544	543	543	403	403	547	547
AIC	9,618.5	9,610.2	8,062.5	8,050.5	683.0	681.4	2,167.0	2,168.2
BIC	9,633.2	9,625.0	8,077.4	8,065.4	695.5	694.0	2,182.0	2,183.2

*z statistics in brackets*.

* *p < 0.10*,

** *p < 0.05*,

*** *p < 0.01*.

*Model 1 is the animal health treatment effect model. Model 2 is the breeding intervention treatment effects model. Fertility denotes the number of lambs/kids born per breeding female in a year. Offtake denotes the number of sheep/goat sold in a year. Ln(return/head) denotes natural log of revenue generated per head of sheep/goat in birr. Ln(income/AE) denotes natural log of gross annual income per adult equivalent in birr. N is number of observations*.

*AIC, Akaike information criterion; BIC, Bayesian information criterion; DiD, difference-in-differences; AE, adult equivalent*.

Although the intervention and control sites were selected randomly based on a very comprehensive characterization effort (6), we considered, based on theory and econometric criteria, literacy in years of education, family size, and distance to livestock market in kilometers as potential confounders of the cause-and-effect relationship (Table 6). This was not however the case, and our estimation simply reinforced the estimator with no covariate reported in Table 5. Participating in community-based veterinary services and small ruminant breeding has increased only offtake rates in the project sites.

**Table 6:** DiD with basic fixed-effects specification with covariates.

	**Fertility**		**Offtake**		**Ln(return/head)**		**Ln(income/AE**)	
	**Model 1**	**Model 2**	**Model 1**	**Model 2**	**Model 1**	**Model 2**	**Model 1**	**Model 2**
Animal health ^*^ Year	−2.40 [−0.42]		5.96^**^ [2.39]		0.44 [1.18]		−0.20 [−1.60]	
Breeding ^*^ Year		−5.38 [−0.90]		8.86^***^ [3.36]		0.35 [0.91]		−0.20 [−1.52]
Year	1.42 [0.42]	0.37 [0.11]	13.56^***^ [8.40]	13.43^***^ [8.76]	0.12 [0.53]	0.19 [0.89]	0.43^***^ [5.02]	0.42^***^ [5.12]
Animal health	1.15 [0.17]		−5.71^**^ [−2.37]		0.10 [0.25]		−0.11 [−0.72]	
Breeding		11.68* [1.79]		−8.10^***^ [−3.33]		−0.04 [−0.11]		−0.06 [−0.49]
Literacy (years)	1.21 [0.36]	0.96 [0.29]	1.64 [1.37]	1.33 [1.08]	0.47 [1.23]	0.48 [1.23]	−0.08 [−1.10]	−0.09 [−1.19]
Family size	1.96 [1.34]	1.69 [1.14]	0.39 [0.78]	0.50 [1.02]	0.32^**^ [2.13]	0.31^**^ [2.07]	−0.07^**^ [−2.43]	−0.07^**^ [−2.33]
Extension on sheep/goat (yes = 1)	2.61 [0.56]	2.18 [0.48]	−0.47 [−0.30]	−0.56 [−0.36]	−0.32 [−1.10]	−0.34 [−1.13]	0.03 [0.40]	0.03 [0.37]
Constant	12.84 [1.10]	12.57 [1.12]	4.98 [1.35]	4.64 [1.26]	1.93* [1.92]	2.05^**^ [2.20]	8.41^***^ [35.58]	8.37^***^ [36.97]
*N*	1,018	1,018	1,042	1,042	483	483	1,081	1,081
*N*_cluster	544.00	544.00	543.00	543.00	403.00	403.00	547.00	547.00
AIC	9,601.63	9,595.10	8,039.33	8,027.65	579.90	587.98	2,152.57	2,154.19
BIC	9,631.18	9,624.65	8,069.02	8,057.34	604.98	613.06	2,182.49	2,184.10

*z statistics in brackets*.

* *p < 0.10*,

** *p < 0.05*,

*** *p < 0.01*.

*Model 1 is the animal health treatment effect model. Model 2 is the breeding intervention treatment effects model. Fertility denotes the number of lambs/kids born per breeding female in a year. Offtake denotes the number of sheep/goat sold in a year. Ln(return/head) denotes natural log of revenue generated per head of sheep/goat in birr. Ln(income/AE) denotes natural log of gross annual income per adult equivalent in birr. N is number of observations*.

*AIC, Akaike information criterion; BIC, Bayesian information criterion; DiD, difference-in-differences; AE, adult equivalent*.

Finally, we report the DID model estimated on the common support formed based on the propensity score. Treatment effect estimations can be improved through joint specification of DiD and PSM based on pretreatment variables. By combining PSM and DiD, in addition to the unobservable time-invariant characteristics, the observable heterogeneity in the initial conditions can be controlled (25, 30). This estimator also helps in checking the robustness of the impacts observed in the FE models presented above (Tables 5, 6).

The DiD–PSM specification that considered the pre-intervention variables^5^ resulted in an enhanced cause-and-effect relationship between participation in community-based veterinary services and small ruminant offtake, revenue per head of sheep/goat, and gross income/AE. Similarly, participation in CBBP has positively and significantly improved small ruminant offtake and gross income/AE (Table 7).

**Table 7:** Treatment effect estimated using DiD with PSM.

	**Fertility**		**Offtake**		**Ln(return/head)**		**Ln(income/AE)**	
	**Model 1**	**Model 2**	**Model 1**	**Model 2**	**Model 1**	**Model 2**	**Model 1**	**Model 2**
Animal health ^**^ Year	−0.59 [−0.15]		17.53^***^ [12.28]		0.59^**^ [2.12]		0.19^**^ [2.57]	
Breeding ^*^ Year		1.16 [0.30]		18.06^***^ [12.23]		0.49 [1.60]		0.19^**^ [2.39]
Literacy (years)	1.21 [0.35]	1.13 [0.33]	0.96 [0.70]	1.20 [0.87]	0.47 [1.21]	0.47 [1.20]	−0.09 [−1.21]	−0.09 [−1.19]
Family size	1.95 [1.33]	1.97 [1.33]	0.45 [0.81]	0.26 [0.46]	0.33^**^ [2.32]	0.33^**^ [2.28]	−0.07^**^ [−2.32]	−0.07^**^ [−2.38]
Extension on sheep/goat (yes = 1)	2.72 [0.59]	2.60 [0.57]	−0.14 [−0.08]	−0.04 [−0.03]	−0.31 [−1.01]	−0.32 [−1.03]	0.05 [0.56]	0.05 [0.56]
Constant	13.56 [1.22]	13.18 [1.17]	6.37 [1.59]	7.54^*^ [1.86]	1.95^**^ [2.08]	2.01^**^ [2.15]	8.46^***^ [35.75]	8.48^***^ [36.15]
*N*	1,018	1,018	1,042	1,042	483	483	1,081	1,081
*N*_cluster	544.00	544.00	543.00	543.00	403.00	403.00	547.00	547.00
AIC	9,597.96	9,597.82	8,209.79	8,219.89	577.39	588.07	2,207.63	2,209.50
BIC	9,617.67	9,617.52	8,229.58	8,239.69	594.11	604.79	2,227.57	2,229.44

*z statistics in brackets*.

* *p < 0.10*,

** *p < 0.05*,

*** *p < 0.01*.

*Model 1 is the animal health treatment effect model. Model 2 is the breeding intervention treatment effects model. Fertility denotes the number of lambs/kids born per breeding female in a year. Offtake denotes the number of sheep/goat sold in a year. Ln(return/head) denotes natural log of revenue generated per head of sheep/goat in birr. Ln(income/AE) denotes natural log of gross annual income per adult equivalent in birr. N is number of observations*.

*AIC, Akaike information criterion; BIC, Bayesian information criterion; DiD, difference-in-differences; PSM, propensity score matching; AE, adult equivalent*.

*The reviewer SS declared a shared affiliation with one of the authors, SG, to the handling editor at time of review*.

Small ruminant keepers participating in veterinary interventions have supplied about 18 more sheep/goat to the market than those who did not participate. These farmers have generated 80.4% higher revenue per head of sheep/goat and 21% more gross income/AE. The farmers who participated in CBBP have also supplied 18 more sheep and goat to the market in a period of 12 months. These farm households also earned 20.6% more gross income/AE than did those who did not participate in the breeding program.

In summary, the estimations we made show that participating in the community-based veterinary and breeding interventions improves market participation in terms of supplying higher number of small ruminants to the market. For participants in veterinary interventions, this higher participation is associated with higher return/animal. This is expected, as pests and diseases are among the most important challenges that small ruminant keepers are facing at every level of the production–consumption continuum. In the markets, for instance, one of the insecurities embedded in livestock transactions is the uncertainty around the health status of the animal. Any intervention that ensures the healthiness of the animals will certainly increase the number of animals the farmers raise and bring to the rural markets (1).

Our findings are in line with other positive contributions of animal health services reported in previous research works. Based on a monitoring study that compared infection with strongyle and *Fasciola* species before and after the a community-based intervention, Gizaw et al. (41) reported that the likelihood of worm infection was significantly lower among livestock after farmers started the collective action for worm control. Admassu et al. (42) similarly observed that there was significant reduction in the impact of diseases handled by community animal health workers (CAHWs) compared with diseases not handled by CAHWs in Ethiopia. Based on simulation study, Beyi (43) reported that milk loss in non-vaccinated dairy herds in Ethiopia was 2.3 and 19.4 times higher than in herds receiving reactive and preventive vaccination against foot and mouth disease, respectively. McDermott et al. (44) and Roth et al. (45) have also reported positive evidence of the returns to investment in brucellosis control, particularly in vaccination of livestock, measured in both livestock productivity and gains in human health.

We also observed that community-based breeding interventions consistently improve small ruminant offtake rates. The higher number of sales is also reflected in the increased gross income per adult equivalent. We have however observed that the return per animal is not statistically different between those who participate in CBBP and those who do not. Yet this is expected as the breeding interventions include identification of the best rams and culling (selling) the ones that do not score high in the traits of interest. In fact, the lack of difference between the participants and non-participants in the breeding interventions could explain the fact that some of the animals are culled at a young age and not necessarily at the right price.

## Conclusion

International and national partners designed and implemented community-based small ruminant breeding and health interventions in carefully selected sites in various parts of Ethiopia since 2014. This study presents an assessment of the impact of these interventions using two waves (2014 and 2018) of survey data. Different specifications of DiD treatment effects modeling were estimated to investigate the impact of these interventions on sheep/goat fertility, offtake rate, revenue per head of sheep/goat, and gross income/AE.

The different estimations show that veterinary and breeding interventions have significantly increased the number of sheep and goats smallholders supply to the market. The most robust estimator that combined PSM and DiD to control for initial conditions has enhanced the causality between the interventions and the outcomes. Those who participated in community-based veterinary services showed higher offtake, higher return per sheep/goat, and higher annual income/AE. Similarly, those who participated in CBBP managed to sell higher number of small ruminants and earned higher aggregate income/AE.

The positive and statistically significant effect of animal health services not only justifies the participatory approach employed in identifying and prioritizing the animal health challenges but also sheds light on to what extent investments on animal health service are rewarding at the community level. The results in this paper shall serve as an additional input to the global appeal for participatory and comprehensive animal health service provision (46–48). Similarly, the results we reported show that the CBBPs designed and implemented by and with the small ruminant keeping community are rewarding in many ways. This is a testament for the argument that introduction of exotic genetic materials is not necessarily the solution for the genetic improvement of the livestock resources of rural communities in Africa or in Ethiopia in particular (10, 49). It is, therefore, imperative to suggest further strengthening and scaling up of the community-based small ruminant breeding programs as long as meaningful financial and economic benefits are to accrue to the society.

We expect the results reported here to inform the national effort that is being exerted in Ethiopia to transform the agricultural sector in general and the livestock sector in particular. Interventions that address the key challenges of the livestock keeping community are crucially important if the livelihoods of the people who depend on their animals are to improve. Proper measurement of the welfare of these interventions helps in prioritizing resource allocation and justifying the interventions at scale.

Finally, we highlight two limitations of our study that can be addressed building on the analytical framework we presented. First, our study covers only sedentary farming systems. Livestock are the mainstay of livelihood in pastoral and agro-pastoral livelihood systems. We anticipate the impacts of livestock services to be more pronounced in these systems. The second limitation is that we focused only on small ruminants. All other species of livestock are equally important in the rural economy of Ethiopia. Broader research will generate empirical evidence that can be used to economically justify prioritization of investments in livestock development across the different production systems.

## Data Availability Statement

The raw data supporting the conclusions of this article will be made available by the authors, without undue reservation.

## Ethics Statement

Ethical review and approval was not required for the study on human participants, in accordance with the local legislation and institutional requirements.

## Author Contributions

GK led the design, data collection, data analysis, and writing the results of the research. WA coordinated the field data collection during the end line survey and led the cleaning of the data. AH contributed in the designing of the study and writing the research article. TM and SG contributed in writing the research article. BR contributed in managing the entire research program and in writing the research article. All authors contributed to the article and approved the submitted version.

## Conflict of Interest

The authors declare that the research was conducted in the absence of any commercial or financial relationships that could be construed as a potential conflict of interest. The reviewer SS declared a shared affiliation with one of the authors, SG, to the handling editor at time of review.

## Publisher's Note

All claims expressed in this article are solely those of the authors and do not necessarily represent those of their affiliated organizations, or those of the publisher, the editors and the reviewers. Any product that may be evaluated in this article, or claim that may be made by its manufacturer, is not guaranteed or endorsed by the publisher.

## Footnotes

^1^Kebele [pl. Kebeles] is the smallest administrative unit in Ethiopia.

^2^Kebele, plural Kebeles, is the smallest unit of administration in Ethiopia.

^3^Some interventions were started before 2014, and some sites were added after the baseline survey was conducted. We are reporting only for sites where the baseline survey was conducted and the interventions that followed were informed by the baseline survey.

^4^Birr is the official currency of Ethiopia, and the average exchange rate in December 2018 was 1 birr = 3.6 US cents.

^5^PSM model results and common support graphs are not reported for brevity reasons. They are available upon request for interested readers.

## References

[1] Kassie GT Wubie RS Tokgoz S Majeed F Yitayih M Rischkowsky B. Policy-induced price distortions along the small ruminant value chains in Ethiopia. J Agribus Dev Emerg Econ. (2019) 9:220–36. 10.1108/JADEE-02-2018-0024

[2] Shapiro BI Gebru G Desta S Negassa A Nigussie K Aboset G. Ethiopia Livestock Sector Analysis, ILRI Project Report. Nairobi: International Livestock Research Institute (ILRI) (2017).

[3] Wodajo HD Gemeda BA Kinati W Mulem AA van Eerdewijk A Wieland B. Contribution of small ruminants to food security for Ethiopian smallholder farmers. Small Rumin Res. (2020) 184:106064. 10.1016/j.smallrumres.2020.106064

[4] Awgichew K Gebru G Alemayehu Z Akalework N Fletcher J. Small ruminant production in Ethiopia: constraints and future prospects. In: Proc. 3rd Natl. Livest. Improv. Conf. Addis Ababa, (1991).

[5] Getachew T Haile A Wurzinger M Rischkowsky B Gizaw S Abebe A. Review of sheep crossbreeding based on exotic sires and among indigenous breeds in the tropics: an Ethiopian perspective. African J Agric Res. (2016) 11:901–11. 10.5897/AJAR2013.10626

[6] Legese G Haile A Duncan A Dessie T Gizaw S Rischkowsky B. Sheep and Goat Value Chains in Ethiopia: A Synthesis of Opportunities and Constraints, ICARDA/ILRI Project Report. Nairobi: ICARDA & ILRI (2014).

[7] Solomon A Grum G Haile A Rischkowsky B Solomon G Dessie T. Review of Sheep Research and Development Projects in Ethiopia. Nairobi: International Livestock Research Institute (ILRI), (2013).

[8] Ayalew W King JM Bruns E Rischkowsky B. Economic evaluation of smallholder subsistence livestock production: lessons from an Ethiopian goat development program. Ecol Econ. (2003) 45:473–85. 10.1016/S0921-8009(03)00098-3

[9] Gizaw S Getachew T Edea Z Mirkena T Duguma G Tibbo M. Characterization of Indigenous Breeding Strategies of the Sheep Farming Communities of Ethiopia: A Basis for Designing Community-Based Breeding Programs, Working Paper. Aleppo: ICARDA (2013).

[10] Haile A Getachew T Mirkena T Duguma G Gizaw S Wurzinger M. Community-based sheep breeding programs generated substantial genetic gains and socioeconomic benefits. Animal. (2020) 14:1362–70. 10.1017/S1751731120000269 32100664 PMC7301245

[11] Haile A Gizaw S Getachew T Mueller JP Amer P Rekik M. Community-based breeding programmes are a viable solution for Ethiopian small ruminant genetic improvement but require public and private investments. J Anim Breeding Genet. (2019) 136:319–28. 10.1111/jbg.12401 31037758

[12] Ministry of Agriculture International Livestock Research Institute. Animal Health Strategy and Vision for Ethiopia. Nairobi: Ministry of Agriculture and ILRI (2013).

[13] Klooster Gvan't. Key Achievements and Strategic Impacts: EU-SHARE PPR Project. Rome: Food and Agriculture Organization of the United Nations (2019).

[14] Silkin T. Veterinary services in the Horn of Africa: where are we now? Dev Pract. (2005) 15:40–8. 10.1080/0961452052000321569

[15] Alemu B Desta H Kinati W Mulema AA Gizaw S Wieland B. Application of mixed methods to identify small ruminant disease priorities in Ethiopia. Front Vet Sci. (2019) 6:417. 10.3389/fvets.2019.00417 32039243 PMC6988793

[16] Adam B Dawit A Tilaye T Henrietta LM Catherine H Stefan B. Strategies for animal disease control in Ethiopia: a review of policies, regulations and actors. J Vet Med Anim Heal. (2018) 10:256–65. 10.5897/JVMAH2018.0711

[17] Marshall K Gibson JP Mwai O Mwacharo JM Haile A Getachew T. Livestock genomics for developing countries – African examples in practice. Front Genet. (2019) 10:297. 10.3389/fgene.2019.00297 31105735 PMC6491883

[18] Mueller JP Rischkowsky B Haile A Philipsson J Mwai O Besbes B. Community-based livestock breeding programmes: essentials and examples. J Anim Breed Genet. (2015) 2015:jbg.12136. 10.1111/jbg.12136 25823840

[19] Catley A Alders RG Wood JLN. Participatory epidemiology: approaches, methods, experiences. Vet J. (2012) 191:151–60. 10.1016/j.tvjl.2011.03.010 21856195

[20] Mekonnen M Assefa A Nane T Ayele F Arke A Elias B. Small Ruminant Health Intervention Calendar in Ethiopia. Nairobi: International Livestock Research Institute (ILRI) (2019).

[21] Gizaw S Desta H Alemu B Tegegne A Wieland B. Importance of livestock diseases identified using participatory epidemiology in the highlands of Ethiopia. Trop Anim Health Prod. (2020) 52:1745–57. 10.1007/s11250-019-02187-4 31898026 PMC7858201

[22] Haile A Wurzinger M Mueller J Mirkena T Duguma G Okeyo Mwai A. Guidelines for Setting Up Community-Based Sheep Breeding Programs in Ethiopia: Lessons and Experiences for Sheep Breeding in Low-Input Systems, ICARDA - Tools and Guidelines. Aleppo: ICARDA (2011).

[23] Caliendo M Kopeinig S. Some practical guidance for the implementation of propensity score matching. J Econ Surv. (2008) 22:31–72. 10.1111/j.1467-6419.2007.00527.x

[24] Gertler PJ Martinez S Premand P Rawlings LB Vermeersch CMJ. Impact Evaluation in Practice. 2nd ed. Washington, DC: Inter-American Development Bank and World Bank (2016). 10.1596/978-1-4648-0779-4

[25] Wing C Simon K Bello-Gomez RA. Designing difference in difference studies: best practices for public health policy research. Ann Rev Public Health. (2018) 39:453–69. 10.1146/annurev-publhealth-040617-013507 29328877

[26] Chakrabarti S Kishore A Roy D. Effectiveness of food subsidies in raising healthy food consumption: public distribution of pulses in India. Am J Agric Econ. (2018) 100:1427–49. 10.1093/ajae/aay022

[27] Crown WH. Propensity-score matching in economic analyses: comparison with regression models, instrumental variables, residual inclusion, differences-in-differences, and decomposition methods. Appl Heal Econ Heal Policy. (2014) 12:7–18. 10.1007/s40258-013-0075-4 24399360

[28] Stuart EA Huskamp HA Duckworth K Simmons J Song Z Chernew ME. Using propensity scores in difference-in-differences models to estimate the effects of a policy change. Heal Serv Outcomes Res Methodol. (2014) 14:166–82. 10.1007/s10742-014-0123-z 25530705 PMC4267761

[29] Villa JM. Diff: simplifying the estimation of difference-in-differences treatment effects. Stata J. (2016) 16:52–71. 10.1177/1536867X1601600108

[30] Ravallion M. Evaluating Anti-Poverty Programs, Evaluating Anti-Poverty Programs. Washington, DC: Elsevier (2008). 10.1016/S1573-4471(07)04059-4

[31] Angrist J Pischke J-S. Mostly Harmless Econometrics: An Empiricist's Companion. New Jersey: Princeton University Press (2009). 10.1515/9781400829828

[32] Morgan SL Winship C. Counterfactuals and Causal Inference: Methods And Principles For Social Research (Analytical Methods for Social Research). 2nd ed. Cambridge: Cambridge University Press (2014). 10.1017/CBO9781107587991

[33] Cameron AC Trivedi PK. Microeconometrics Using Stata. Revised ed. Texas: Stata Press (2010).

[34] Strumpf EC Harper S Kaufman JS. Fixed effects and difference-in-differences. In: Oakes JM Kaufman JS editors, Methods in Social Epidemiology. San Francisco, CA: Jossey-Bass (2017).

[35] Dimick JB Ryan AM. Methods for evaluating changes in health care policy: the difference-in-differences approach. J Am Med Assoc. (2014) 312:2401–2. .org/10.1001/jama.2014.16153 25490331 10.1001/jama.2014.16153

[36] Khandker S Koolwal G Samad H. Handbook on Impact Evaluation. Washington, DC: World Bank. (2009). 10.1596/978-0-8213-8028-4

[37] Abadie A. Semiparametric difference-in-differences estimators. Rev Econ Stud. (2005) 72:1–19. 10.1111/0034-6527.00321

[38] Zeleke F Kassie GT Haji J Legesse B. Preference and willingness to pay for small ruminant market facilities in the central highlands of Ethiopia. J Int Food Agribus Mark. (2020) 1–23. 10.1080/08974438.2020.1838385

[39] uo S Fraser MW. Propensity Score Analysis: Statistical Methods and Applications. 2nd ed. Thousand Oaks, CA: Sage (2015).

[40] CSA. The 2015/16 Ethiopian Household Consumption – Expenditure (HCE) Survey: Results for country level, Statistical Report. Addis Ababa: Central Statistical Agency (2018).

[41] Gizaw S Desta H Berhanu D Barbara W. A Narrative Review of Animal Health Interventions for Designing Herd Health Interventions for Ethiopia. Addis Ababa: International Livestock Research Institute. (2019).

[42] Admassu B Nega S Haile T Abera B Hussein A Catley A. Impact assessment of a community-based animal health project in Dollo Ado and Dollo Bay districts, southern Ethiopia. Trop Anim Health Prod. (2005) 37:33–48. 10.1023/B:TROP.0000047932.70025.44 15729896

[43] Beyi AF. Feed the Future Innovation Lab for Livestock Systems, Ethiopia: Animal Source Foods Production and Marketing Brief. The Management Entity at the University of Florida. Gainesville, FL: University of Florida. (2016).

[44] McDermott J Grace D Zinsstag J. Economics of brucellosis impact and control in low-income countries. OIE Rev Sci Tech. (2013) 32:249–61. 10.20506/rst.32.1.2197 23837382

[45] Roth F Zinsstag J Orkhon D Chimed-Ochir G Hutton G Cosivi O. Human health benefits from livestock vaccination for brucellosis: case study. Bull World Health Organ. (2003) 81:867–76. 14997239 PMC2572379

[46] Perry B Grace D. The impacts of livestock diseases and their control on growth and development processes that are pro-poor. Philos Trans R Soc Lond B Biol Sci. (2009) 364:2643–55. 10.1098/rstb.2009.0097 19687035 PMC2865091

[47] Perry B Sones K. Science for development. Poverty Reduction Through Animal Health Science. (2007) 315:333–4. 10.1126/science.1138614 17234933

[48] Perry BD Rich KM. Poverty impacts of foot-and-mouth disease and the poverty reduction implications of its control. Vet Rec. (2007) 160:238–41. 10.1136/vr.160.7.238 17308024

[49] Kosgey IS Baker RL Udo HMJ Van Arendonk JAM. Successes and failures of small ruminant breeding programmes in the tropics: a review. Small Rumin Res. (2006) 61:13–28. 10.1016/j.smallrumres.2005.01.003

